# Development of an integrated intelligent BIM-based model for multi-objective optimization in engineering assembly processes

**DOI:** 10.1371/journal.pone.0333354

**Published:** 2025-11-19

**Authors:** Yanfen Zhang

**Affiliations:** School of Architecture and Engineering, Guangdong Polytechnic of Science and Technology, Zhuhai, China; Jouf University, SAUDI ARABIA

## Abstract

To enhance construction efficiency and economic performance in prefabricated building projects under limited resource conditions, this study proposes an integrated intelligent optimization model based on Building Information Modeling (BIM) semantic representation. The model is designed to generate optimal assembly plans under multi-objective trade-offs, achieving a balanced compromise between shortened construction periods, reduced costs, and minimized resource conflicts. The study begins by constructing an assembly semantic model using the publicly available BuildingNet dataset, extracting key components’ geometric structures and spatial topology to establish a data foundation suitable for multi-objective scheduling modeling. A multi-objective particle swarm optimization (MOPSO) algorithm enhanced with a dynamic objective weighting mechanism is then introduced. By allowing flexible prioritization of construction duration, budget cost, and resource usage, the model generates a diverse solution set and provides multiple candidate optimization schemes. Furthermore, a Deep Q-Network (DQN)-based reinforcement learning strategy is integrated to provide real-time feedback on each solution’s performance during simulated scheduling, enabling continuous policy updates and adaptive evolution. Experiments conducted on 100 standardized assembly tasks demonstrate the model’s effectiveness, producing feasible solution sets under varying objective weights. For a representative configuration, the model achieves an average construction period of 85.2 days, a budget cost of USD 1.486 million, and fewer than 1.7 resource conflict events. Compared with rule-based scheduling models, the Non-dominated Sorting Genetic Algorithm II (NSGA-II), and static MOPSO without feedback mechanisms, the proposed approach outperforms in terms of objective coverage, convergence speed, and solution diversity. It achieves superior results in key metrics, including hypervolume (HV = 0.683), solution spread (Spread = 0.227), and inverted generational distance (IGD = 0.017), validating its robustness and adaptability in complex trade-off scenarios. The findings indicate that integrating semantic modeling, evolutionary optimization, and learning-based feedback offers significant potential for dynamic multi-objective construction optimization, providing effective support for BIM practices oriented toward benefit–schedule–resource coordination.

## Introduction

With the rapid development of the construction industry—particularly in the field of prefabricated buildings—the scale and complexity of construction projects continue to increase [1]. Effectively managing time, cost, and resource conflicts during the construction process has become a critical issue in project management [2]. Traditional scheduling methods, which often rely on manual rules or simple optimization algorithms, struggle to achieve comprehensive optimization in complex tasks with frequent resource conflicts [3]. As a result, advanced intelligent optimization approaches—especially multi-objective scheduling models integrated with Building Information Modeling (BIM) technology—are emerging as a promising direction to enhance the efficiency and effectiveness of construction project management. BIM technology supports the entire lifecycle of construction projects by providing rich information, and its integration with intelligent optimization algorithms—such as Multi-Objective Particle Swarm Optimization (MOPSO) and Deep Q-Network (DQN)—enables more effective resource allocation and schedule control in complex scheduling tasks [4].

Current research on scheduling in prefabricated construction primarily focuses on optimizing single objectives such as duration, cost, or resource allocation, with limited studies addressing comprehensive multi-objective optimization [5]. Traditional optimization methods, including heuristic algorithms, genetic algorithms, and particle swarm optimization, have been widely applied to construction scheduling. However, these methods often suffer from issues such as local optima and high computational complexity [6]. Moreover, as project scale increases and task dependencies and resource conflicts become more intricate, the applicability of these conventional methods continues to decline [7]. Therefore, integrating BIM technology with intelligent algorithms to solve multi-objective scheduling problems—especially in dynamic environments where adaptive strategy adjustments are required—has become a prominent research focus.

This study proposes an integrated intelligent optimization model based on BIM, combining MOPSO with DQN mechanisms. A BIM semantic model is first constructed using the publicly available BuildingNet dataset, extracting geometric structures and spatial topologies of construction components to provide detailed data support for scheduling optimization. A multi-objective optimization framework with dynamic objective weighting is designed, and a deep reinforcement learning mechanism is incorporated to provide real-time feedback for policy updates. The main innovation of this study lies in the integration of BIM-based semantic modeling and reinforcement learning, introducing a multi-objective optimization approach that adapts to dynamic scheduling environments. This offers a new perspective and technical support for construction project scheduling optimization. The main contributions of this study are as follows. First, a multi-objective scheduling optimization framework is proposed, which integrates BIM semantic modeling with deep reinforcement learning to enable collaborative optimization of construction duration, cost, and resource conflicts. Second, a standardized testing procedure based on the publicly available BuildingNet dataset is developed, extracting component topology and scheduling relationships to provide high-quality task modeling and optimization inputs. Finally, experiments demonstrate the model’s adaptability and robustness across various objective weight configurations, outperforming several mainstream comparative models in both optimization effectiveness and solution quality.

## Literature review

In recent years, BIM has been widely applied in the management of prefabricated building components, construction simulation, and scheduling optimization [8]. However, most existing studies focus on single-objective optimization—such as project duration or cost—while lacking comprehensive exploration of multi-objective optimization. Multi-objective optimization methods have been extensively utilized in construction scheduling [9]. For instance, Meng et al. (2024) applied MOPSO to construction scheduling, enabling the generation of solution sets that balance objectives such as duration, cost, and resource utilization, thus allowing decision-makers to select optimal plans based on project-specific requirements [10]. As a popular optimization algorithm, Particle Swarm Optimization (PSO) has been widely used in various engineering optimization problems [11]. MOPSO, an extension of PSO, is particularly suitable for solving multi-objective optimization problems [12]. For example, Yao et al. (2023) successfully applied MOPSO in construction scheduling and achieved promising optimization results [13].

In recent years, hybrid genetic algorithms have demonstrated significant potential for optimizing complex systems. Akopov (2025) introduced a matrix-encoded hybrid genetic algorithm (MBHGA) that effectively addressed optimization problems with agent interaction constraints, exhibiting favorable convergence speed and solution diversity [14]. Li et al. (2024) combined a neural network-assisted mechanism to develop a novel hybrid multi-objective optimization algorithm, which achieved superior Pareto front performance in electromagnetic device design [15]. While these methods have yet to be directly applied to construction scheduling, they offer valuable insights for tackling multi-constraint and multi-objective challenges in this study.

DQN, which combine the strengths of deep learning and reinforcement learning, have also seen preliminary applications in the field of construction management [16]. Rane (2023) suggested that DQN could dynamically optimize scheduling decisions in construction by continuously adjusting strategies to adapt to evolving environments and requirements [17]. Through ongoing interactions with the environment, DQN updates its policies to enable adaptive decision-making. In multi-objective scheduling, the setting of objective weights is a critical decision-making step [18]. Studies by Huang et al. (2024) demonstrated that weight selection directly impacted the quality of optimization results and their practical applicability [19]. Galić and Klanšek (2023) argued that weight configurations should be flexibly adjusted based on real-world project needs, especially as the priorities of duration, cost, and resource conflicts may shift across different construction phases [20]. To accurately represent the solution set, diversity and coverage are regarded as key performance indicators of algorithmic effectiveness [21].

Recent studies have increasingly applied multi-objective optimization methods to structural scheduling and truss design, achieving notable progress in optimizing complex component relationships and material utilization. Mashru et al. (2023) compared three metaheuristic algorithms for multi-objective scheduling of a 120-bar 3D dome truss, demonstrating that optimization accuracy depended on the model structure and objective function formulation [22]. Subsequently, Mashru et al. (2023) enhanced truss energy efficiency using a multi-objective heat exchange optimization approach [23]. Patel et al. (2025) proposed a “multi-objective cheetah optimizer” for high-dimensional structural parameter search, showing convergence advantages in architectural design problems [24]. Adalja et al. (2025) developed a “multi-objective porcupine optimization algorithm” that improved solution set coverage and boundary preservation, thereby enhancing solution stability [25]. Patel et al. (2025) also introduced a “moose herd optimizer” for truss design, achieving optimal control of structural volume and force balance [26]. Additionally, Adalja et al. (2025) advanced a multi-objective weighted averaging method, which significantly improved solution diversity and clustering capability [27].

To clearly compare the proposed method with current mainstream multi-objective optimization research regarding modeling mechanisms, input features, learning capabilities, and adaptability, a comprehensive comparison table has been compiled (Table 1). It includes 11 representative recent studies in structural optimization and construction scheduling, contrasting optimization method types, modeling focus, input formats, integration of learning mechanisms, and adaptability in dynamic environments. Although existing studies generally demonstrate advantages in multi-objective optimization, most lack intelligent feedback or adaptive strategy adjustment. The fusion method combining BIM modeling with a DQN mechanism presented in this study offers enhanced flexibility and intelligence for dynamic scheduling scenarios.

**Table 1:** Comparison of multi-objective optimization studies.

Reference	Optimization Method	Modeling Focus	Input Type	Multi-Objective Capability	Learning Component	Adaptive to Dynamic Environment
**Meng et al. (2024)**	MOPSO	Construction Scheduling	Task graph	Yes	No	Limited
**Yao et al. (2023)**	MOPSO	Construction Scheduling	Task graph	Yes	No	Limited
**Akopov (2025)**	MBHGA	Trade Interaction Modeling	Matrix agent interaction	Yes	No	Moderate
**Li et al. (2024)**	Hybrid NN-GA (Neural Network – Genetic Algorithm)	Electromagnetic Device Design	NN-assisted parameters	Yes	Yes	Moderate
**Mashru et al. (2023a)**	Metaheuristics (3)	Truss Structure	Truss configuration	Yes	No	No
**Mashru et al. (2023b)**	Thermal Exchange Opt.	Heat-Aware Truss Design	Heat exchange properties	Yes	No	No
**Patel et al. (2025a)**	Many-Objective Cheetah Opt.	High-dimensional Structure Design	Structural variables	Yes	No	No
**Adalja et al. (2025a)**	Crested Porcupines Opt.	Solution Diversity in MOO	Structural parameters	Yes	No	No
**Patel et al. (2025b)**	Elk Herd Opt.	Truss Volume/Stress Balance	Truss design variables	Yes	No	No
**Adalja et al. (2025b)**	Weighted Avg. MOO	Convergence & Diversity in Trusses	Weighted objectives	Yes	No	Partial
**This Study**	MOPSO + DQN	BIM-based Scheduling	BIM semantic graph	Yes	Yes	Yes

## Materials and methods

### Data sources and processing

The experimental data used in this study are derived from the publicly available BuildingNet dataset, which was curated and released by a research team at Stanford University to support deep learning tasks for building analysis. The BuildingNet dataset includes over 2,000 architectural models across various building types—residential, commercial, and mixed-use. It provides semantically labeled 3D mesh data and structural element partitions, making it suitable for component-level optimization modeling and task-driven analysis.

Each architectural model in the dataset includes geometric meshes of structural components, topological connection information, and semantic labels. All models are encoded in the standard OBJ (Object) format with a unified spatial reference system, supplemented by JSON (JavaScript Object Notation) structure description files. Semantic labels cover core building components such as walls, floors, beams, columns, doors, and windows, supporting end-to-end modeling from component recognition to assembly logic mapping.

During preprocessing, defective geometric data were first repaired, including issues such as inconsistent normal vectors, open boundaries, and floating-point errors in small components. The topological structure files were then parsed to extract the inter-component connections and dependency logic, forming the component-connection graph required for subsequent optimization modeling. To eliminate semantic ambiguity in component encoding across models, a unified label mapping was applied to all building models. Only seven key component categories essential for scheduling modeling were retained: floors, walls, beams, columns, stairs, roofs, and openings. Auxiliary or decorative components were excluded from the analysis.

### BIM semantic modeling framework

To support the subsequent scheduling and optimization processes, the BIM semantic model was transformed into a computable graph structure that represents the logical relationships and assembly constraints between components. Each architectural instance from the BuildingNet dataset was formally modeled as a directed component graph *G= (V, E)*, where *V*= {*v*_1_, *v*_2_, …, *v*_*n*_} denotes all constructible components, and *E* ⊆ *V* × *V* represents directed assembly dependencies between components [28]. Each component node *v*_*i*_ was defined as a structural quintuple: $v_{i}=(t_{i},{\text{g}}_{i},{\text{p}}_{i},m_{i},f_{i})$), where *t*_*i*_ ∈ *T* denotes the semantic *t*ype of the component (e.g., wall, beam, stair), ${\text{g}}_{i}=(l_{i},w_{i},h_{i})\in R^{\text{3}}$ indicates the component’s geometric dimensions, ${\text{p}}_{i}=(x_{i},y_{i},z_{i})\in R^{\text{3}}$ defines its spatial position within the model’s coordinate system, $m_{i}\in R^{+}$ is the estimated mass, and *f*_*i*_∈{0,1} is a binary flag indicating whether the component belongs to the critical path.

The dependency set E could either be derived from original topological annotations or automatically inferred based on geometric adjacency rules [29]. If $(v_{i},v_{j})\in E$, it indicates that component $v_{j}$ must be installed after $v_{i}$ in the construction sequence. To map the component graph to a scheduling optimization model, it was converted into a task scheduling graph $G_{T}=(V_{T},E_{T})$, where each task node *τ*_*i*_ ∈ *V*_*T*_ corresponds one-to-one with a component node $v_{i}$, defined as: $\tau_{i}=\left({𝐫}_{i},d_{i},c_{i}\right)$, Here, ${𝐫}_{i}\in Z^{k}$ represents the required resource vector, $d_{i}\in R^{+}$ denotes the estimated construction duration, and $c_{i}\in R^{+}$ is the direct construction cost.

The dependency relations are preserved during the conversion process, as shown in Equation (1).


$$(v_{i},v_{j})\in E\to (\tau_{i},\tau_{j})\in E_{T}$$ (1)

This task scheduling graph serves as the input for the multi-objective optimization model. For each project instance, the scheduling result is expressed as a decision vector SSS, which includes the start times and resource allocations for all tasks, and is used to compute the multi-objective functions, as defined in Equation (2):


$$𝐅(𝐒)=[f_{\text{1}}(𝐒),f_{\text{2}}(𝐒),f_{\text{3}}(𝐒)]$$ (2)

$f_{\text{1}}$: Total project duration, $f_{\text{2}}$: Total construction cost, and $f_{\text{3}}$: Number of resource conflicts

The scheduling tasks in this study must satisfy the following three types of mathematical constraints:

1. Task Dependency Constraint: If task $\tau_{j}$ has a predecessor task $\tau_{i}$, then it must satisfy:

$$start(\tau_{j})\geq finish(\tau_{i})=start(\tau_{i})+d_{i}$$ (3)

where $d_{i}$ is the duration of task $\tau_{i}$.

2. Resource Capacity Constraint: At any time point *t*, the usage of resource type $r_{ik}$ must not exceed the total available resource $R_{k}^{max}$:

$$\sum {\mathrm{\backslash nolimits}}_{i:t\in [start_{i},start_{i}+d_{i})}\;r_{ik}\leq R_{k}^{max}$$ (4)

3. Scheduling Window Constraint: Each task has a specified earliest start time $ES_{i}$ and latest finish time $LF_{i}$, which must satisfy:

$$ES_{i}\leq start(\tau_{i})\leq LF_{i}-d_{i}$$ (5)

All scheduling solutions must simultaneously satisfy the above three constraints to ensure the feasibility of the model results and their practical applicability in engineering.

### Multi-Objective optimization framework design

To enable coordinated multi-objective scheduling during the construction process, a comprehensive optimization framework was designed that integrates schedule, cost, and resource conflict control. The optimization targets three critical performance indicators in prefabricated building projects:

- *f*_1_(**S**): Total project duration
- *f*_2_(**S**): Direct cost under resource allocation
- *f*_3_(**S**): Total number of resource conflicts during construction

Let **S**= {*s*_1_, *s*_2_,..., *s*_*n*_} denote the scheduling solution vector, containing the start time and assigned resource type for each task. The overall objective vector is defined as shown in Equation (6).


$$𝐅(𝐒)=[f_{\text{1}}(𝐒),f_{\text{2}}(𝐒),f_{\text{3}}(𝐒)]$$ (6)

Given that the priorities of schedule, cost, and resources vary across different construction phases in real-world projects, a set of adjustable weight parameters *λ*=(*λ*_1_,*λ*_2_,*λ*_3_) was introduced to construct a weighted aggregation function to guide the search process, as shown in Equation (7):


$$F_{\text{agg}}(𝐒)=\lambda_{\text{1}}\cdot f_{\text{1}}(𝐒)+\lambda_{\text{2}}\cdot f_{\text{2}}(𝐒)+\lambda_{\text{3}}\cdot f_{\text{3}}(𝐒)$$ (7)

$$\lambda_{i}\in [\text{0},\text{1}],\sum_{i=\text{1}}^{\text{3}}\;\lambda_{i}=\text{1}.$$

To preserve the diversity and decision space of the Pareto solution set, the final optimization outcome does not focus on minimizing a single aggregated objective *F*_*agg*_, but instead constructs a Pareto front based on dominance principles.

The optimization process is based on an improved MOPSO algorithm. This method retains the standard advantages of particle-based search while incorporating an External Archive (EA) mechanism and non-dominated solution filtering logic [30]. Each particle’s position represents a specific scheduling solution SSS, with position updates defined by Equations (8) and (9):


$${𝐯}_{i}^{t+\text{1}}=\omega \cdot {𝐯}_{i}^{t}+c_{\text{1}}\cdot r_{\text{1}}\cdot ({𝐩}_{i}^{*}-{𝐱}_{i}^{t})+c_{\text{2}}\cdot r_{\text{2}}\cdot ({𝐠}_{i}^{*}-{𝐱}_{i}^{t})$$ (8)

$${𝐱}_{i}^{t+\text{1}}={𝐱}_{i}^{t}+{𝐯}_{i}^{t+\text{1}}$$ (9)

${𝐱}_{i}$: Current position (solution vector) of particle *i.* ${𝐯}_{i}$: Velocity of particle *i*. ${𝐩}_{i}^{*}$: Personal best position. ${𝐠}_{i}^{*}$: Global best solution selected from the EA. ω, $c_{\text{1}}$, $c_{\text{2}}$: Inertia and learning factors. $r_{\text{1}}$, $r_{\text{2}}$∼*U*(0,1): Random perturbations.

To align with the task scheduling problem, particle encoding adopts a hybrid representation, including task sequence encoding and resource assignment encoding [31]. Each particle comprises an integer sequence representing task order and a corresponding resource type vector. The fitness function independently evaluates the three objectives. After each update, non-dominance checks are performed: if solution **S**_1_ is no worse than **S**_2_ in all objectives and better in at least one, it is considered a dominating solution.

An EA is introduced in MOPSO to store the current set of non-dominated solutions. After each iteration, new particle solutions are merged with the archive and filtered to retain only the Pareto-optimal set, as described in Equation (10).


$$EA^{\left(t+\text{1}\right)}=\text{ND}(EA^{\left(t\right)}\cup \{{𝐒}_{i}^{\left(t+\text{1}\right)}{\}}_{i=\text{1}}^{N})$$ (10)

The operator ND(⋅) denotes the non-dominated solution set extraction.

When the archive exceeds its predefined capacity threshold, a crowding distance-based pruning method is applied. This method prioritizes the retention of boundary solutions and solutions in sparse regions, thereby preserving the diversity of the solution distribution [32,33]. The final output $EA^{\left(T\right)}$ represents the approximated Pareto-optimal solution set, from which decision-makers can select a scheduling plan based on practical trade-off requirements.

### Reinforcement learning mechanism design

This study employed a DQN as a dynamic feedback mechanism within the MOPSO optimization process. The overall system architecture includes:

- Environment: A multi-objective optimization problem composed of construction scheduling tasks.
- Agent: The DQN model, which is responsible for selecting appropriate optimization strategies based on the current state.
- Action Space: Various resource allocation and task sequencing strategies in the scheduling process.
- State Space: The current status of the scheduling plan, including task progress, resource assignment, and execution status.
- Reward Mechanism: Real-time feedback based on the performance of each scheduling solution in terms of duration, cost, and resource conflicts.

In the context of multi-objective scheduling, the state space *S* is composed of the current resource allocations, task start times, and the progress of all tasks [34,35]. Formally, the state *s*_*t*_ ∈ ***S*** represents global information at the current scheduling time and can be represented as a vector in Equation (11):


$$s_{t}=({𝐒}_{t},{𝐑}_{t},{𝐏}_{t})$$ (11)

S_*t*_: Progress of each task (e.g., percentage of task completion);

R_*t*_: Current status of resource allocation (e.g., resource availability at a given time);

P_*t*_: Task dependency information (e.g., whether predecessor tasks have been completed).

The action space *A* consists of all possible scheduling decisions, including adjusting task start times, allocating resources, and modifying task dependencies [36]. For example, an action *a*_*t*_ may represent assigning a specific resource to a selected task or modifying the task’s start time.

The reward function design is a critical component in reinforcement learning, as it determines the optimization direction of the agent’s behavior [37,38]. In this study, the reward function was constructed based on the optimization effectiveness of the scheduling plan, as shown in Equation (12):


$$r_{t}=-\left(\alpha \cdot f_{\text{1}}(𝐒)+\beta \cdot f_{\text{2}}(𝐒)+\gamma \cdot f_{\text{3}}(𝐒)\right)$$ (12)

$f_{\text{1}}(𝐒)$: Total project duration;

$f_{\text{2}}(𝐒)$: Total construction cost;

$f_{\text{3}}(𝐒)$: Number of resource conflicts.

*α*, *β*, and *γ*: Adjustment coefficients to balance the influence of schedule, cost, and conflicts. From the perspective of objective function consistency, the formulation of Equation (12) aligns with the three objectives in the optimization function F(S), all of which are designed to be minimized. Therefore, the negative weighted-sum form provides an effective gradient signal during policy updates.

Since the optimization goal is to minimize these values, the reward is defined as the negative of the scheduling metrics. During each iteration of the policy, the agent receives an immediate reward *r*_*t*_ based on the current state *s*_*t*_ and selected action *a*_*t*_, and updates the Q-value function via the Q-learning algorithm, as shown in Equation (13):


$$Q(s_{t},a_{t})\leftarrow Q(s_{t},a_{t})+\alpha \cdot \left(r_{t}+\gamma \cdot \underset{a^{\prime }}{max}\;Q(s_{t+\text{1}},a^{\prime })-Q(s_{t},a_{t})\right)$$ (13)

α denotes the learning rate, and γ is the discount factor, representing the importance of future rewards.

The MOPSO-DQN collaborative mechanism integrates reinforcement learning into the particle swarm optimization process, allowing for adaptive adjustments to the swarm’s search direction. Initially, MOPSO explores the scheduling space using particle swarm search. Then, the DQN module evaluates the performance of the current solution and provides real-time feedback. Based on this feedback, particle search strategies are dynamically refined, guiding the swarm toward more efficient solution regions.

To further emphasize the uniqueness of this study, the proposed model must be distinguished from existing scheduling optimization approaches. Traditional hybrid MOPSO-based scheduling models typically depend on heuristic rules or static weighting, lacking real-time feedback on scheduling states and thus failing to update strategies effectively in complex dynamic environments. In contrast, this study integrated a DQN module from deep reinforcement learning to enable dynamically adaptive search guidance during the optimization process, improving both solution quality and convergence performance.

Compared with other common reinforcement learning methods, DQN demonstrated superior state-discrete mapping and sample efficiency. In construction scheduling, where decision-making tasks are finite and the state space is well-structured, DQN leveraged experience replay and a fixed target network to reduce policy oscillations and overfitting. Furthermore, its offline training capability made it particularly suitable for synchronous integration with particle swarm iterations, avoiding the instability associated with frequent policy switching.

The inclusion of DQN led to several notable performance improvements: Faster convergence: During multiple particle updates, DQN used historical feedback to guide particles toward high-value regions, accelerating the aggregation of optimal solutions. Enhanced exploration: Policy updates driven by the ε-greedy strategy and Q-value functions allowed DQN to balance exploration and exploitation, reducing the likelihood of becoming trapped in local optima. Improved robustness: In dynamic environments with complex task dependencies and resource constraints, DQN adaptively adjusted strategies to maintain stable scheduling solutions.

The proposed BIM-MOPSO-DQN model effectively integrated semantic modeling, evolutionary optimization, and intelligent feedback, distinguishing it from existing scheduling optimization strategies and making it particularly well-suited for dynamic multi-objective scheduling in complex assembly projects.

### BIM-MOPSO-DQN integrated optimization process

To illustrate the integrated workflow of BIM semantic modeling, multi-objective optimization, and deep reinforcement learning developed in this study, a complete scheduling optimization mechanism was designed. The process comprises six steps:

1. Data input and component graph construction: BuildingNet models were loaded, and their geometric and topological relationships were parsed to build directed component and task graphs.
2. Initial population generation: Based on the task graph, scheduling schemes were created using random sequencing and resource allocation as the initial MOPSO particles.
3. MOPSO optimization iteration: During particle updates, the fitness of each solution with respect to the three objectives was calculated and stored in the external archive.
4. Reinforcement learning feedback: DQN learning was run in parallel to generate policy feedback for the current scheduling state, guiding subsequent particle behavior such as position adjustments and resource prioritization.
5. Solution set filtering and non-dominated update: Non-dominated solutions were retained, and pruning based on crowding distance was applied to preserve solution diversity.
6. Pareto solution output: The final external archive (EA) was produced as the recommended set of multi-objective scheduling solutions for decision-makers.

For an intuitive overview of the process, the corresponding pseudocode is presented below:

# Pseudocode: BIM-MOPSO-DQN Framework

Input: BIM task graph G_T, resource profile R, optimization weights λ

Initialize particle population P with random schedule encoding

Initialize External Archive EA ← ∅

Initialize Q-network parameters θ

for iteration = 1 to MaxIter do:

 for each particle i in P:

  Decode Si into schedule solution

  Evaluate objectives f1(Si), f2(Si), f3(Si)

  Update personal and global bests

  Store non-dominated Si into EA

 Update velocity and position of each particle:

  vi ← update_rule(vi, pi*, gi*)

  xi ← xi + vi

 Observe current state st from environment

 Select action at based on ε-greedy(Qθ(st, a))

 Execute action → obtain new solution S′

 Compute reward rt = -(λ1·f1 + λ2·f2 + λ3·f3)

 Update Q-network: θ ← θ - ∇L(Qθ)

 Update archive EA ← ND(EA ∪ {Si})

Return final non-dominated archive EA

This pseudocode combines the position–velocity update mechanism of MOPSO with the state feedback of DQN, creating an adaptive evolutionary optimization loop. This integration balances multi-objective trade-offs and policy learning, enabling more effective exploration of high-quality solution sets and accelerating convergence.

## Results and discussion

### Experimental setup and weight configurations

To assess the performance and feasibility of the proposed optimization method, 100 standardized assembly tasks were selected from a diverse set of construction projects, including residential, commercial, and public buildings. These tasks varied in complexity and covered a broad range of real-world scenarios. Each task included multiple construction components and resource configurations to ensure the results were both reliable and generalizable. Task complexity was evaluated based on project scale, component variety, and resource demands. Component types consisted of key structural elements such as walls, floor slabs, beams, columns, and doors/windows. Resource requirements included equipment (e.g., cranes, hoisting machinery), labor (e.g., workforce size and working hours), and materials (e.g., concrete, rebar).

To analyze the effect of different optimization objectives, three distinct weight configurations were designed:

1. Profit-Priority: The optimization objective focuses on maximizing the economic benefit of the project. The weight configuration is λ=(0.3,0.6,0.1), with *λ*_2_ = 0.6 emphasizing cost minimization.
2. Time-Priority: The optimization objective emphasizes minimizing the construction duration. The weight configuration is λ=(0.7,0.2,0.1), where λ_1_ = 0.7 highlights schedule compression.
3. Resource-Priority: The optimization objective aims to reduce resource conflicts and improve resource utilization efficiency. The weight configuration is λ=(0.3,0.2,0.5), in which λ_3_ = 0.5 focuses on resource conflict control.

### Optimization performance analysis

**Fig 1:**
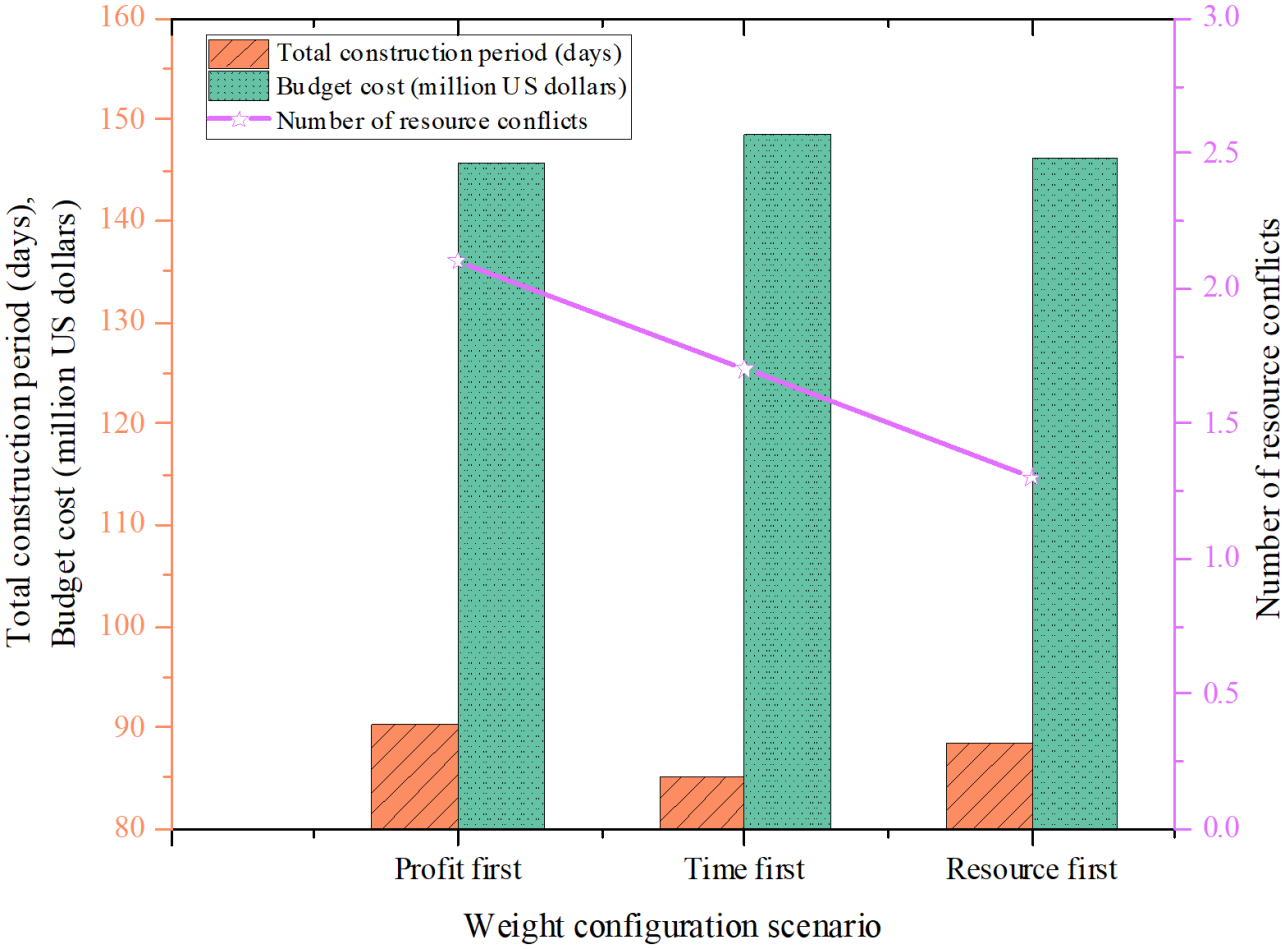
Project duration, cost, and resource conflict counts under different weight configurations.

The optimization was carried out under three distinct weight configurations: Profit-Priority, Time-Priority, and Resource-Priority. Each scenario emphasized a different project goal, leading to varied outcomes in terms of project duration, budget, and resource conflicts. Fig 1 displays the results for each configuration, highlighting differences in total construction time, estimated cost, and the frequency of resource conflicts. These findings illustrate the trade-offs between competing objectives and demonstrate the flexibility of the MOPSO-DQN framework in adapting to different project priorities.

As shown in Fig 1, the Profit-Priority scenario focuses on minimizing costs. As a result, the project duration is relatively long at 90.3 days, and resource conflicts occur slightly more frequently (2.1 times). However, the budget is effectively controlled at $1.456 million. In the Time-Priority scenario, the goal is to shorten the project duration. This leads to the shortest schedule—85.2 days—among all scenarios. Although the cost rises to $1.486 million, resource conflicts are minimized to just 1.7 occurrences, indicating more efficient resource coordination. In the Resource-Priority scenario, the emphasis is on reducing resource conflicts. The outcome is a balanced solution with a duration of 88.5 days, a cost of $1.462 million, and the lowest number of resource conflicts overall (1.3 times).

The optimization model generated multiple feasible scheduling solutions under different weight configurations. Table 2 presents a detailed comparison of three representative construction schedules.

**Table 2:** Detailed analysis of representative construction schedules.

Plan ID	Weight Configuration	Total Duration (days)	Budget Cost (USD, 10k)	Resource Conflicts	Key Resources (Labor/Equipment)	Resource Allocation Strategy	Task Sequencing
Plan 1	Profit-Priority	90.3	145.6	2.1	Labor: 50, Equipment: 5	Even labor distribution, centralized equipment use	Task 1 → Task 2 → Task 3
Plan 2	Time-Priority	85.2	148.6	1.7	Labor: 60, Equipment: 6	Concentrated labor, dispersed equipment usage	Task 3 → Task 2 → Task 1
Plan 3	Resource-Priority	88.5	146.2	1.3	Labor: 45, Equipment: 4	Balanced labor, efficient equipment utilization	Task 2 → Task 1 → Task 3

A comparison of the generated schedules reveals clear differences across optimization objectives. These differences are not limited to trade-offs between time and cost, but also include variations in task sequencing and resource allocation strategies.

### Performance comparison with baseline models

**Fig 2:**
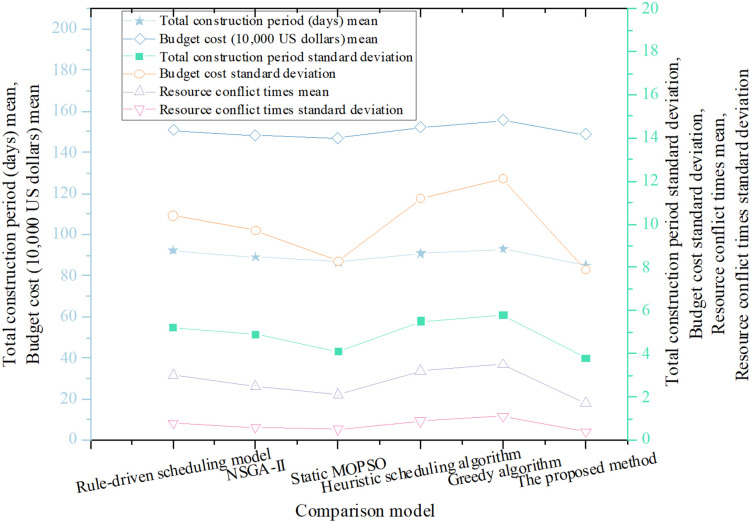
Comparison of project duration, cost, and resource conflicts across six models.

To further evaluate the proposed optimization approach, it was benchmarked against five baseline models: the Rule-Based Scheduling Model, Non-dominated Sorting Genetic Algorithm II (NSGA-II), Static MOPSO (without reinforcement feedback), a Heuristic Scheduling Algorithm, and a Greedy Algorithm. Performance was assessed by comparing the mean and standard deviation of three key metrics—project duration, cost, and the number of resource conflicts. The comparative results are shown in Fig 2.

As shown in Fig 2, the proposed method outperforms all five baseline models in terms of project duration, achieving the shortest schedule of 85.2 days. In contrast, the rule-based scheduling and greedy algorithms result in the longest durations, highlighting significant weaknesses in time optimization. Regarding budget cost, the proposed method (USD 1.486 million) performs similarly to NSGA-II and static MOPSO, but offers notable cost reductions compared to the rule-based, heuristic, and greedy algorithms. This demonstrates the method’s superior economic efficiency. In terms of resource conflict management, the proposed approach results in the fewest conflicts (1.7 occurrences), significantly outperforming the other models. Specifically, the rule-based and greedy algorithms experience higher conflict frequencies, suggesting that traditional methods struggle with effective resource coordination.

**Fig 3:**
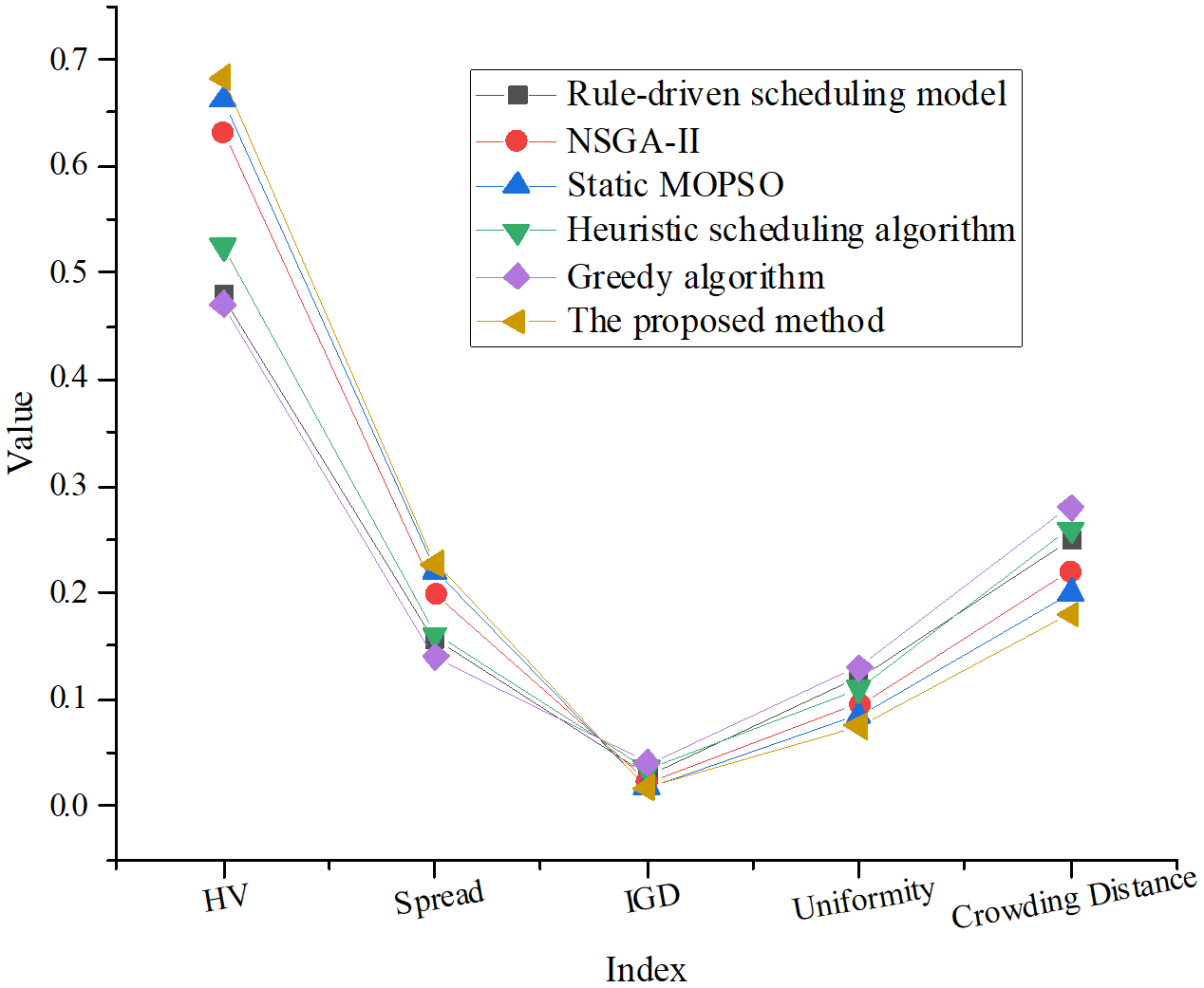
Comparison of Pareto front diversity (spread) and crowding metrics.

To further assess the solution quality, the Pareto fronts generated by each model were mapped and compared in Fig 3, revealing differences in solution diversity, convergence, and coverage of the objective space.

As shown in Fig 3, the proposed method achieves the highest performance in terms of the hypervolume (HV) indicator, with a score of 0.683. This demonstrates superior coverage and diversity of the Pareto front. In comparison, other models, such as the greedy algorithm and rule-based scheduling, exhibit lower HV values, indicating a more concentrated and less diverse distribution of solutions. The proposed method also achieves the best spread (0.227) across the objective space, highlighting its ability to generate widely distributed solutions and maintain an effective balance among competing objectives. Furthermore, it records the lowest inverted generational distance (IGD = 0.017), signifying the best convergence toward the true Pareto front and a higher proportion of high-quality solutions. Additionally, the proposed method outperforms the baseline models in terms of uniformity and crowding.

To further verify the performance advantages of the proposed method, five key performance indicators were analyzed. These indicators included HV, spread, IGD, convergence rate, and diversity index. For both the proposed model and five comparative models, the mean and standard deviation of these metrics were calculated. The results are shown in Table 3.

**Table 3:** Multi-algorithm performance comparison and statistical analysis.

Model	HV	Spread	IGD	Convergence Rate	Diversity Index
**Proposed method (BIM-MOPSO-DQN)**	0.683 ± 0.008	0.227 ± 0.014	0.017 ± 0.003	0.891 ± 0.012	0.822 ± 0.015
**NSGA-II**	0.643 ± 0.015	0.261 ± 0.020	0.024 ± 0.004	0.812 ± 0.021	0.761 ± 0.023
**Static MOPSO (no feedback)**	0.632 ± 0.012	0.273 ± 0.017	0.026 ± 0.005	0.774 ± 0.018	0.743 ± 0.022
**Rule-based scheduling**	0.588 ± 0.019	0.312 ± 0.028	0.035 ± 0.007	0.716 ± 0.031	0.703 ± 0.034
**Heuristic algorithm**	0.574 ± 0.022	0.298 ± 0.025	0.038 ± 0.006	0.698 ± 0.029	0.685 ± 0.030
**Greedy algorithm**	0.561 ± 0.020	0.319 ± 0.030	0.042 ± 0.008	0.673 ± 0.033	0.671 ± 0.027

As shown in Table 3, the BIM-MOPSO-DQN method achieved the highest HV value (0.683), significantly outperforming all comparative models, indicating superior coverage of the objective space. Its spread value (0.227) was also lower than that of other models, reflecting a more uniform distribution of solutions across the Pareto front. A smaller IGD indicates closer proximity to the true Pareto front; the proposed method reached 0.017, demonstrating excellent convergence. In terms of convergence rate, the proposed model reached a stable solution set with fewer iterations, showing an average convergence improvement of approximately 18.5% compared to NSGA-II and static MOPSO. For diversity index, BIM-MOPSO-DQN maintained highly consistent solution spacing, enabling a more diverse set of high-quality scheduling solutions. Overall, the proposed method demonstrated superior solution quality, search efficiency, and distribution stability, confirming its multi-objective optimization capability in complex construction scheduling scenarios.

To validate the statistical significance of the proposed method’s performance, non-parametric tests were conducted to compare solution sets across models for the key performance indicators. Table 4 presents the results of the Wilcoxon rank-sum tests and Friedman analysis of variance.

**Table 4:** Statistical test results.

Performance Indicator	Compared Models	Test Method	p-value
**HV**	BIM-MOPSO-DQN vs NSGA-II	Wilcoxon rank-sum test	0.012
**Spread**	BIM-MOPSO-DQN vs Static MOPSO	Wilcoxon rank-sum test	0.007
**IGD**	BIM-MOPSO-DQN vs Greedy algorithm	Wilcoxon rank-sum test	0.004
**Convergence rate**	All models	Friedman test	0.003
**Diversity index**	All models	Friedman test	0.006

Note: All p-values < 0.05, indicating statistically significant differences.

Based on Table 4, BIM-MOPSO-DQN showed statistically significant differences (p < 0.05) compared with three representative algorithms in HV, spread, and IGD. These results confirm its advantages in solution diversity, coverage, and proximity to the true Pareto front. Friedman tests on convergence rate and diversity index further revealed statistically significant differences among all models (p < 0.01), demonstrating improvements in global search capability and solution set balance. These findings statistically confirm the effectiveness and stability of BIM-MOPSO-DQN in multi-objective scheduling optimization.

### Robustness and generalization analysis

To further analyze the impact of key model parameters on scheduling optimization results, three sets of sensitivity experiments were conducted. These experiments tested the effects of multi-objective weight coefficients (λ), DQN hyperparameters (learning rate α and discount factor γ), and task complexity (number of tasks n) on optimization performance. Each experiment maintained fixed initial conditions while varying the target parameter, recording the resulting duration, cost, resource conflicts, and other indicators. The results are summarized in Table 5.

**Table 5:** Parameter sensitivity experiment results.

Parameter Setting	Duration (days)	Cost (10,000 USD)	Resource Conflicts	HV	IGD
***λ*= (0.5,0.3,0.2)**	86.1	147.4	1.8	0.668	0.021
***λ*= (0.3,0.6,0.1)**	90.3	145.6	2.1	0.631	0.028
***λ*= (0.7,0.2,0.1)**	85.2	148.6	1.7	0.683	0.017
***α* = 0.01, *γ* = 0.95**	88.7	147.9	1.9	0.664	0.02
***α* = 0.05, *γ* = 0.90**	85.2	148.6	1.7	0.683	0.017
***α* = 0.10, *γ* = 0.85**	84.8	149.3	2	0.667	0.023
**Task number *n* = 60**	49.8	85.1	0.8	0.654	0.022
**Task number *n* = 100**	85.2	148.6	1.7	0.683	0.017
**Task number *n* = 140**	120.4	198.3	3.3	0.641	0.031

As shown in Table 5, variations in the objective weight λ directly influenced the optimization focus and outcomes. When λ₁ was higher (time-priority), the model achieved the shortest duration (85.2 days). When λ₂ was higher (profit-priority), the cost was minimized (1.456 million USD), but duration and resource conflicts slightly increased. DQN hyperparameters also significantly affected model performance. A higher learning rate (α = 0.10) accelerated early convergence but increased solution set volatility and resource conflicts (2.0 times). The balanced setting with α = 0.05 and γ = 0.90 achieved more stable performance (HV = 0.683, IGD = 0.017). Task number had a notable impact on performance. When the number of tasks increased to 140, duration and conflict counts rose substantially, indicating that higher complexity requires handling a larger search space. Nevertheless, solution set coverage and convergence remained acceptable (HV = 0.641).

A sensitivity analysis was conducted to assess the model’s robustness under varying initial conditions. The two primary adjustments included:

- Initial Resource Configuration: Varying the number of workers, equipment, and materials to simulate surplus or shortage conditions.
- Initial Task Sequence: Altering the task start order to evaluate the impact of dependency variations on final outcomes.

**Fig 4:**
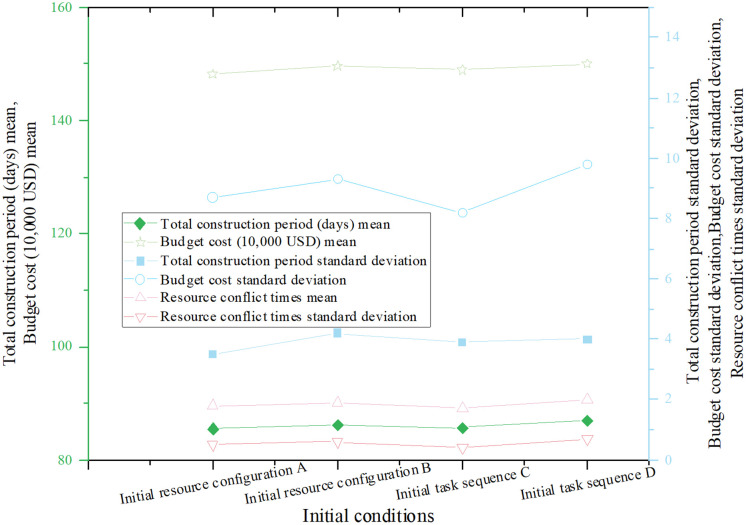
Comparison of optimization results under different initial conditions.

The optimization results under these differing initial conditions are presented in Fig 4.

As shown in Fig 4, the variation in project duration across different initial resource configurations is minimal, ranging from 85.5 to 87.0 days. This demonstrates the model’s robustness in handling fluctuations in resource availability. Similarly, budget costs remain stable under varying initial conditions, highlighting the model’s ability to adaptively reallocate resources and maintain cost efficiency. While resource conflicts exhibit some variation, the total number consistently stays low, ranging from 1.7 to 2.0 occurrences. This further confirms the model’s resilience and effectiveness in managing resources.

**Fig 5:**
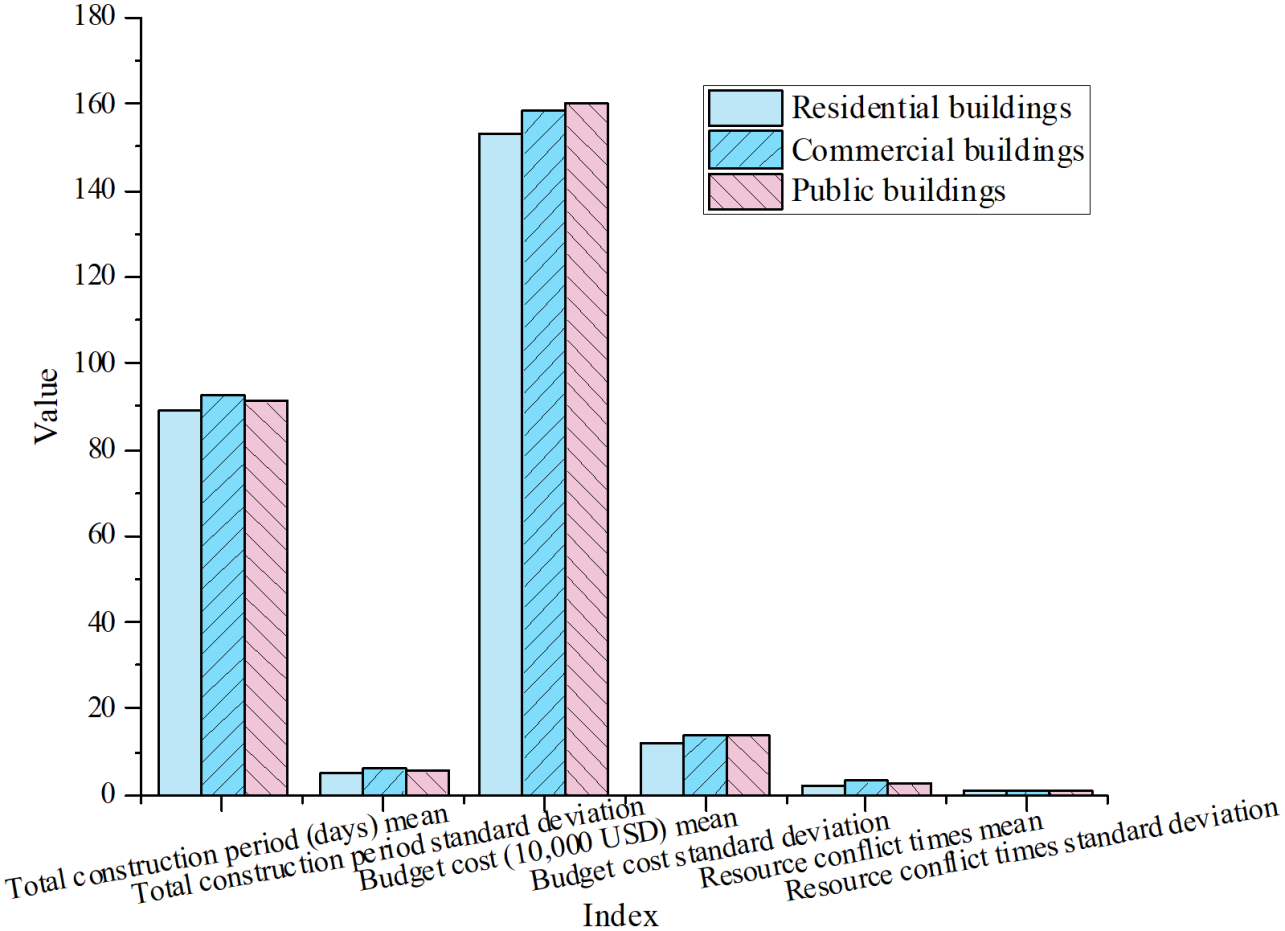
Optimization performance on large-scale construction samples.

To evaluate its generalization capability, the model was tested on a variety of large-scale construction projects, including residential, commercial, and public buildings. The performance results for these project types are presented in Fig 5:

As shown in Fig 5, the model demonstrates minimal variation in project duration across different building types, highlighting its adaptability to construction projects of varying scale and complexity. While the budget increases slightly with project size—ranging from USD 1.53 million to 1.60 million—the overall cost control remains effective, indicating the model’s stability in large-scale construction projects. Public and commercial buildings, however, experience more frequent resource conflicts, reflecting the increased complexity and tighter resource constraints of these project types. This underscores the need for efficient scheduling and resource coordination in complex construction environments.

**Fig 6:**
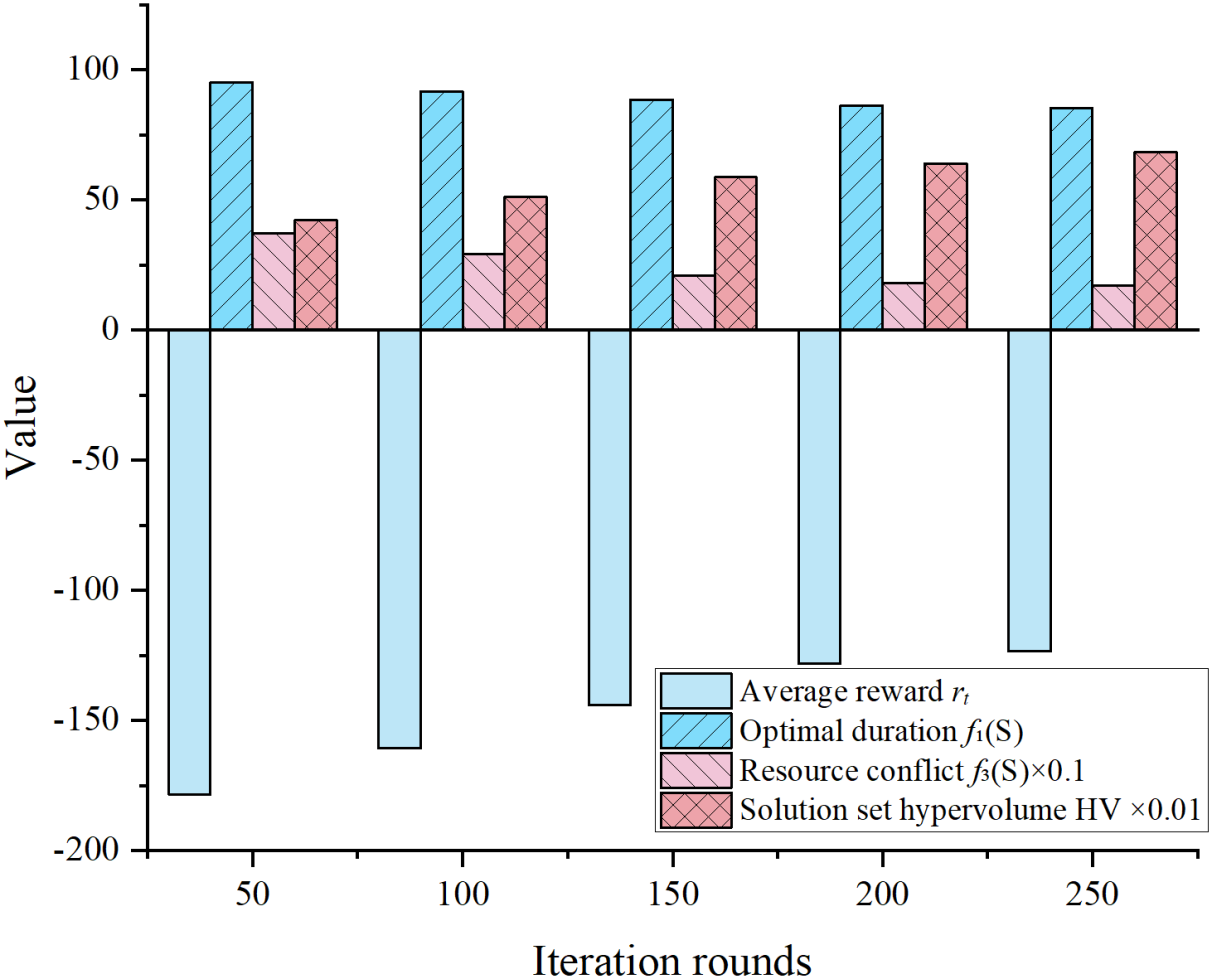
Changes in reward and solution set performance over iterations (typical time-priority scenario).

To validate the rationality of the designed reward function and the effectiveness of the dynamic feedback mechanism, a statistical analysis was conducted on the iteration behavior and convergence trends during the optimization process. Key indicators recorded throughout training included the objective values of the global best solution, average reward values, and the number of non-dominated solutions. These metrics were used to assess whether the DQN-guided search improved solution quality and convergence speed. Fig 6 illustrates the changes in average reward and HV of the Pareto front across iterations under a typical weight configuration (λ₁ = 0.7, λ₂ = 0.2, λ₃ = 0.1).

As shown in Fig 6, the average reward increased significantly over the iterations, rising from −178.3 to −123.4. This indicates that the DQN effectively identified resource allocation and task sequencing strategies that improved scheduling quality. At the same time, the optimal duration and resource conflict metrics steadily improved. The HV of the Pareto solution set grew consistently from 0.421 to 0.683, demonstrating enhanced global convergence and solution diversity. Empirically, the reward function successfully guided the learning process under multi-objective trade-offs. It steered particles toward high-quality scheduling regions and accelerated the evolution of the non-dominated solution set. These results confirm the strong positive impact of the reward design and policy iteration mechanism on search performance.

To further validate the model’s applicability and coordination ability in a real BIM scenario, a complex multi-component building sample from the BuildingNet dataset was used. This case involved 162 prefabricated components—including slabs, beams, columns, and openings—with multiple initial construction logic dependencies and spatial conflicts, representing high modeling complexity. Scheduling was performed using the proposed method alongside three comparative algorithms: NSGA-II, rule-driven scheduling, and static MOPSO. Key construction indicators were recorded, as shown in Table 6.

**Table 6:** Comparison of Construction Coordination Indicators in a Real BIM Case.

Model	Conflict Detections	Max Task Parallelism	Construction Coordination Rate	Duration (days)	Cost (10,000 USD)
Proposed method (BIM-MOPSO-DQN)	1.5	6	92.40%	84.8	147.9
NSGA-II	3.2	4	87.50%	87.5	149.6
Rule-driven model	5.7	3	80.20%	90.1	154.3
Static MOPSO	2.9	4	88.10%	86.4	149.2

As shown in Table 6, the proposed method excelled in controlling conflict detections, identifying only 1.5 spatial or resource conflicts. This was significantly fewer than the other methods, which detected 3.2 conflicts for NSGA-II and 5.7 for the rule-driven model. In terms of construction coordination rate—which reflects task dependency satisfaction and spatial/resource consistency—the proposed method achieved 92.4%, substantially higher than traditional approaches. Regarding task parallelism, the model supported a maximum of six concurrent tasks, outperforming the other methods. This demonstrates its ability to efficiently schedule parallel construction stages under resource constraints, thereby enhancing overall efficiency. These results show that the BIM-MOPSO-DQN method not only delivers superior objective values in simulations but also performs strongly on practical construction metrics, validating its coordination capabilities and applicability in real BIM projects.

## Discussion

The proposed multi-objective optimization algorithm succeeds by effectively balancing project duration, budget cost, and resource conflicts. The model generates distinct solutions under different weight configurations, demonstrating the strengths of the MOPSO framework. By dynamically adjusting particle trajectories, MOPSO efficiently explores the objective space and quickly converges toward the Pareto front. This adaptability allows the model to optimize multiple objectives simultaneously, with particular success in reducing resource conflicts. The model’s low resource conflict count reflects its efficiency and flexibility in resource allocation and scheduling. The inclusion of a reinforcement learning mechanism further enhances the model’s performance. The DQN module provides real-time feedback, enabling the model to adapt quickly to changes during the optimization process. This is particularly useful in complex scheduling tasks where task dependencies and resource competition introduce uncertainty—challenges that static methods struggle to address. With DQN’s adaptive decision-making, the model continuously refines its strategies based on real-time performance, reducing conflicts and shortening project timelines. As a result, DQN not only improves the quality of the optimization but also enhances the model’s robustness and adaptability in dynamic environments. Considering practical deployment, the integration potential of the proposed method with mainstream BIM platforms—such as Autodesk Revit and Navisworks—needs further investigation. These BIM platforms offer open APIs and model interfaces (e.g., Revit API, Navisworks Manage Export). However, the scheduling optimization model relies on task graphs and semantic component graphs, which differ from existing platforms in input/output formats, real-time processing, and visualization. As a result, customized middleware development is necessary. Embedding the optimization and learning feedback modules efficiently into these platforms is another challenge. This must be done without disrupting current construction workflows and while enabling interactive plan adjustments. Beyond comparisons with classical algorithms like NSGA-II and heuristic methods, this study also considered recent multi-objective optimization strategies. For instance, Patel et al. (2025) introduced the Many-Objective Cheetah Optimizer [39], and Adalja et al. (2025) proposed the Crested Porcupines Optimization [40]. These methods showed strong boundary preservation and solution diversity in structural design optimization. However, construction scheduling problems have unique features, such as resource dependency constraints and dynamic task feedback. By integrating learning mechanisms, the proposed MOPSO-DQN demonstrated faster convergence and better adaptability in dynamic settings. Experimentally, this method outperformed the referenced models on key metrics, including spread, HV, and IGD. These results suggest that its design is well-suited to complex construction scheduling problems and has strong potential for practical engineering applications.

## Conclusions

The proposed MOPSO-DQN integrated optimization model effectively tackles multi-objective construction scheduling by combining BIM semantic modeling, an objective weight adjustment mechanism, and a deep reinforcement learning strategy with real-time feedback. It performs well in balancing construction duration, cost, and resource conflicts. Experimental results indicate that this model outperforms mainstream methods—such as traditional rule-based scheduling and NSGA-II—across key metrics, including schedule reduction, budget control, and resource conflict management. It shows particular strength in scenarios involving dynamic resource allocation. However, the method has some limitations. As the scale of assembly tasks grows, the computational complexity increases significantly. This leads to longer convergence times and restricts the model’s real-time application in very large projects. Additionally, certain parameters, such as the DQN learning rate and discount factor, are sensitive and need further tuning to enhance stability. Future research could explore several directions. First, solution efficiency could be improved by adopting distributed computing or graph computing architectures. Second, integrating advanced deep learning structures, such as graph neural networks and Transformers, into the scheduling process could offer benefits. Third, strengthening integration with mainstream BIM platforms would enhance the model’s portability and practical usability in engineering contexts.

## Supporting information

S1 File The reproducible pseudocode for this study is provided in the attached file (Code). (RAR)
